# The antifungal plant defensin AhPDF1.1b is a beneficial factor involved in adaptive response to zinc overload when it is expressed in yeast cells

**DOI:** 10.1002/mbo3.248

**Published:** 2015-03-08

**Authors:** Oriane Mith, Asma Benhamdi, Teddy Castillo, Muriel Bergé, Colin W MacDiarmid, Janet Steffen, David J Eide, Véronique Perrier, Maeva Subileau, Françoise Gosti, Pierre Berthomieu, Laurence Marquès

**Affiliations:** 1 INRA/CNRS UMR B&PMP, Biochimie et Physiologie Moléculaire des Plantes, Montpellier SupAgro/Université Montpellier 2Campus Montpellier SupAgro, 2 Place Viala, F-34060, Montpellier Cedex 2, France; 2 Department of Nutritional Sciences, University of Wisconsin-MadisonMadison, Wisconsin, 53706; 3 INRA/CIRAD UMR 1028 IATE Ingénierie des Agropolymères et Technologies Emergentes, Montpellier SupAgro/Université Montpellier 22 Place Viala, F-34060, Montpellier Cedex 2, France

**Keywords:** Antifungal protein, cellular adaptive response, multifunctionality, plant defensin, zinc tolerance

## Abstract

Antimicrobial peptides represent an expanding family of peptides involved in innate immunity of many living organisms. They show an amazing diversity in their sequence, structure, and mechanism of action. Among them, plant defensins are renowned for their antifungal activity but various side activities have also been described. Usually, a new biological role is reported along with the discovery of a new defensin and it is thus not clear if this multifunctionality exists at the family level or at the peptide level. We previously showed that the plant defensin AhPDF1.1b exhibits an unexpected role by conferring zinc tolerance to yeast and plant cells. In this paper, we further explored this activity using different yeast genetic backgrounds: especially the *zrc1* mutant and an UPRE-GFP reporter yeast strain. We showed that AhPDF1.1b interferes with adaptive cell response in the endoplasmic reticulum to confer cellular zinc tolerance. We thus highlighted that, depending on its cellular localization, AhPDF1.1b exerts quite separate activities: when it is applied exogenously, it is a toxin against fungal and also root cells, but when it is expressed in yeast cells, it is a peptide that modulates the cellular adaptive response to zinc overload.

## Introduction

Antimicrobial peptides are important components of the ancestral innate immune system and are found in all organisms (Maroti et al. 2011). A great diversity of peptides has been found and thousands of sequences are now available. They are subgrouped according to their structure or enrichment in specific amino acids. Among them, plant defensins represent a well-defined subgroup belonging to the large cysteine-rich protein family and sharing a common scaffold based on a typical cysteine-stabilized *α-*helix *β-*sheet motif involving eight cysteines, one *α-*helix, three antiparallel *β-*sheets conferring a very steady structure (van der Weerden and Anderson 2013), and a *γ*-core motif involved in the antifungal activity (Yount and Yeaman 2004; Munoz et al. 2014).

Several plant defensins have been found, but many are yet to be described. Three hundred “defensin-like” genes were forecast in the genome of *Arabidopsis thaliana* (Silverstein et al. 2005). Despite their common structure, a high divergence in their amino acid composition has been found. Numerous plant defensin genes are arranged into clusters suggesting that their diversity arose due to duplication events followed by active divergent selection (Maroti et al. 2011).

A large repertoire of plant defensins is thus available, but very few of these proteins have been studied at the functional level. Up to now, almost all the described plant defensins have shown an antifungal activity (van der Weerden and Anderson 2013). Beside their common structure, this antifungal activity is the hallmark of the plant defensin group. Regarding the mechanism of their antifungal activity, there is no true consensus except for an initial interaction between defensins and sphingolipids (Thevissen et al. 2000, 2004; Ramamoorthy et al. 2007). After this step, several scenarios have been described according to the defensin tested. RsAFP2 and HsAFP1 were reported to generate reactive oxygen species and to induce programmed cell death (Aerts et al. 2007, 2009, 2011). MsDef1 appeared to activate a MAP-kinase signaling cascade (Ramamoorthy et al. 2007), while NaD1 or PsD1 were shown to enter the cell and to exert their effects via some as yet unknown internal targets (Lobo et al. 2007; van der Weerden et al. 2008; Sagaram et al. 2013).

In addition to their antifungal activities, some defensins display other unrelated biological activities recently reviewed by Carvalho and Gomes (Carvalho Ade and Gomes 2009). The modes of action of these side-activities are even less understood except for the *α*-amylase inhibitory activity. This activity is shared by several plant defensins from different species: sorghum (Bloch and Richardson 1991), barley (Mendez et al. 1990), mung bean (Chen et al. 2002), cowpea (Pelegrini et al. 2008). This inhibition was shown to require a steric interaction between the defensin and the enzyme active site (Lin et al. 2007; Pelegrini et al. 2008).

To highlight some relationships between sequences and functions, an attempt to associate phylogenetic clustering and bioactivity of several plant defensins has recently been conducted (van der Weerden and Anderson 2013). This study opens the possibility to predict the multiple activities of plant defensins. However, this approach is hindered by the fact that most new side functions have been discovered along with the description of new plant defensins. No single study has been conducted to test several side functions for the same plant defensin. Indeed, the study of biological activity usually requires either to extract sufficient amounts of purified plant defensin or to produce recombinant native-like defensin, which is far more difficult than simply cloning and expressing the sequence of a new defensin. Given the number of plant defensins described to date, the inventory of their biological activities is daunting. Furthermore, an emerging idea is that plant defensins are multifunctional proteins (Franco 2011) and that, if their mode of action is still not fully clear, it is because their biological activities are diverse and are probably the basis of several modes of action (Sagaram et al. 2011; Munoz et al. 2014). This multiplicity of activities along with the diversity of proteins existing even within a same species adds a new level of complexity.

We previously found that the AhPDF1.1b defensin is antifungal and also confers zinc tolerance to yeast and plant cells (Mirouze et al. 2006; Shahzad et al. 2013). The antifungal activity was tested with the recombinant native-like AhPDF1.1b added externally to fungal cultures, whereas the zinc tolerance was observed when the AhPDF1.1b defensin was expressed in the cell. Unexpectedly, when AhPDF1.1b is expressed in the yeast or in plant leaves, it is not secreted but it accumulates in internal compartments of the secretory pathway (Oomen et al. 2011). When expressed in yeast or in the plant leaf, AhPDF1.1b has thus the same subcellular localization and the same action. We have, therefore, taken advantage of these previous results to use yeast as a cellular model in order to acquire a deeper understanding of the mode of action of AhPDF1.1b. We showed that, when it is applied exogenously, fully oxidized/folded AhPDF1.1b is a toxin for fungal and root cells, while, when it is expressed in yeast cell and retained in the secretory pathway, it contributes to the cellular adaptive response and helps to overcome zinc overload bypassing the tonoplastic transporter ZRC1 that is the primary means to buffer zinc excess.

## Experimental Procedures

### Production of recombinant AhPDF1.1b in *Pichia pastoris*

#### Construction and selection of the *P. pastoris* recombinant clones

Recombinant AhPDF1.1b was produced in *P. pastoris*. To avoid codon usage bias, an optimization of AhPDF1.1b sequence has been done using the overlap extension (SOEing) PCR-based method (Vallejo et al. 1994) (Data S1). The resulting sequence was cloned in the pPICZ*α*C vector between the *Xho*I and *Xba*I sites in frame with the *α*-factor secretion signal from *Sacchomyces cerevisiae*, and under the control of the AOX1 promoter, following the instructions of the manufacturer (Invitrogen, Life Technologies SAS, Saint Aubin, France). The recombinant plasmids were then introduced in *Escherichia coli* DH5*α*™ by heat shock transformation and the recombinant clones were selected on low-salt LB plates (10 g.L^−1^ tryptone, 5 g.L^−1^ yeast extract, 10 g.L^−1^ NaCl, and 15 g.L^−1^ agar) containing 25 mg.L^−1^ Zeocin™. The constructs were checked by sequencing. After having introduced 5 *μ*g of the linearized plasmids into *P. pastoris* X-33 using the lithium chloride transformation method as described in the Invitrogen protocol, cells were grown and selected on YPD plates (10 g.L^−1^ yeast extract, 20 g.L^−1^ peptone, 20 g.L^−1^ glucose, 15 g.L^−1^ agar) containing 0.2 g.L^−1^ Zeocin™. Recombinant clones were then cultured in YPD and 5 *μ*L of the yeast cultures were dropped on YPD plates containing increasing concentrations of Zeocin™ (0.2, 1.2, 1.5, 1.9, and 2.1 g.L^−1^) to select the more tolerant clones.

#### Production of the recombinant AhPDF1.1b in a bioreactor

One of the more Zeocin-resistant clones was cultivated in a bioreactor as previously described (Brunel et al. 2004). Briefly, three successive 24 h precultures under aerobic conditions at 30°C were performed: the first one in YPD medium and the last two in a buffered synthetic medium with glycerol. One hundred milliliters of the last one were used to inoculate 500 mL of glycerol synthetic medium in an Applikon bioreactor of 1 L capacity. Three successive batches on glycerol were conducted to reach a high cell density culture before the induction of the recombinant AhPDF1.1b production by methanol over 5 days.

#### Purification of the recombinant AhPDF1.1b

The AhPDF1.1b was secreted by *Pichia* and thus purified directly from the culture medium. After centrifugation, the culture medium was collected and then successively filtrated at 0.45 and 0.2 *μ*m and further submitted to a diafiltration against 25 mmol/L sodium acetate buffer at pH 5 (buffer A) at a 3 kDa cutoff on a Quixstand system (GE Healthcare, Chalfont St Giles, UK). The diafiltrate was loaded on a cation exchange Streamline SP gel (74 mL, GE Healthcare) equilibrated with buffer A at a flow rate of 10 mL.min^−1^ and washed with buffer A for 1 h. Bound proteins were then eluted at 5 mL.min^−1^ with a ionic gradient starting with 100% buffer B (50 mmol/L Tris-HCl pH 7.6) and ending with 100% of buffer C (1 mol/L NaCl dissolved in buffer B) during 30 min. The elution of proteins was monitored by UV detection (280 and 320 nm). The protein-containing fractions were analyzed by SDS-PAGE and western blot (Data S2). The positive fractions were pooled and diafiltrated on 3-kDa Amicon® Ultra system (Amicon, Millipore, Molsheim, FRANCE). A MALDI-TOF analysis was performed to verify the molecular mass of the produced protein (Data S2). Proteins were then lyophilized and preserved at −20°C for further experiments.

### Antifungal activity against *Fusarium oxysporum* sp. *melonii*

Liquid growth inhibition assays were conducted in a microplate using half strength Potato Dextrose Broth containing a *Fusarium oxysporum* sp*. melonii* spore suspension at 10^4^ spores.mL^−1^ and 10 *μ*g.mL^−1^ tetracycline as previously described (Marquès et al. 2009). To observe the hyphae, the spore suspension was grown with a sublethal concentration of AhPDF1.1b (0.8 *μ*mol/L) and visualized with an Olympus BX61 microscope under bright field.

### Root growth inhibition assay

Root growth inhibition assay was conducted as described by Allen et al. (2008). The surface-sterilized seeds of *A. thaliana* ecotype *Landsberg erecta* were sown in a 24-well sterile assay plate in twice diluted Murashige and Skoog culture medium containing 2% sucrose, 100 mmol/L MES pH 5.6 and supplemented with 0, 4, 8, or 10 *μ*mol/L purified recombinant AhPDF1.1b. The culture was incubated with gentle shaking at 25°C, 16 h photoperiod, for 8 days.

### Expression of AhPDF1.1b and its variants in the yeast *S. cerevisiae*, and tolerance assays toward zinc overload and oxidative treatments

The entire coding sequence, including the signal peptide, of *AhPDF1.1b* was cloned between the *Eco*RI and *Xho*I restriction sites of the URA3+ pYX212 yeast expression vector downstream the constitutive triose phosphate isomerase promoter (Mirouze et al. 2006; Shahzad et al. 2013). A modified *AhPDF1.1b* harboring a SEKDEL-encoding motif fused to the end of its coding sequence was generated from *AhPDF1.1b* by PCR, using the following forward (5′-AAAGAATTCATGGCTAAGTTTGCTTCCATC-3′) and reverse (5′-CCCCTCGAGTTACAATTCATCTTTTTCAGAACATGGGAAGTAACAGATAC-3′) primers. A modified *AhPDF1.1b* coding sequence in which the Cys_15_ and Cys_36_ codons of the mature AhPDF1.1b were changed to alanine codons, was generated by SOEing PCR using the primers given in Data S3. The PCR fragment was digested by the *Eco*RI and *Xho*I restriction enzymes and cloned in pYX212 at the corresponding sites.

All the recombinant constructs were introduced into the BY4741 *S. cerevisiae* strain (MATa, his3Δ1, leu2Δ0, met15Δ0, ura3Δ0) together with the HIS3+ pFL38H vector using the lithium acetate/single-stranded carrier DNA/polyethylene glycol method (Gietz and Woods 2002).

Assaying zinc tolerance was performed in three different ways in the yeast *S. cerevisiae*. Firstly, drop tests were performed on agar selective modified YNB medium, as previously described (Shahzad et al. 2013). Secondly, growth of isolated yeast colonies was also analyzed, starting from 100 *μ*L of a *A*_600 nm_ = 0.01 yeast solution homogeneously plated on a Ø 9 cm petri dish containing the selective modified YNB medium. Pictures of the isolated colonies were taken with a SZX16 Olympus stereozoom microscope and the area of colonies was measured using Image J software (Schneider et al. 2012). Finally, a zinc shock assay was performed on the *zrc1* mutant background according to a protocol previously described (MacDiarmid et al. 2003). Briefly, 5 mL of a *A*_600 nm_ = 0.01 yeast solution made zinc limited by cultivation on low zinc medium (LZM) supplemented with 1 *μ*mol/L zinc during 20 h. The zinc-deficient cells were pelleted, washed, and then inoculated at *A*_600 nm_ = 0.01 in a liquid chelex-treated synthetic-defined (CSD) medium supplemented with 3.5 *μ*mol/L zinc. Growth was followed by *A*_600 nm_ measurement.

The tolerance of yeast cells to oxidative stresses was evaluated in drop tests performed as described previously (Shahzad et al. 2013) except that standard YNB medium (Bio101, Inc., Vista, CA, USA) was used supplemented either with 2.5 mmol/L diamide, 4 mmol/L H_2_O_2_, or 5 *μ*mol/L menadione. All the growth tests were performed at 30°C.

### UPRE-GFP UPR-reporter assay

The UPRE-GFP TEF2-Cherry UPR-reporter *S. cerevisiae* strain (his3Δ1, leu2Δ0, lys2+, met15Δ0, ura3Δ0, UPRE-GFP::URA3, TEF2-cherry::MET15, can1Δ::STE2pr-spHIS5, lyp1Δ::STE3pr-LEU2, cyh2) set up by Jonikas (Jonikas et al. 2009) was kindly given by Dr. Maya Schuldiner. Briefly, a synthetic UPR-responsive promoter composed of four repeats of the *KAR2* UPR-responsive element (UPRE) has been associated with a green fluorescent protein (GFP) reporter gene to monitor unfolded protein response (UPR) activity in vivo. The entire coding sequence of *AhPDF1.1b* was cloned at the *Not*I restriction site of the pFL38H yeast expression vector downstream the constitutive phosphoglycerate kinase 1 promoter. The empty and the recombinant pFL38H vectors were then separately introduced into the WT UPRE-GFP TEF2-Cherry strain using the lithium acetate procedure (Gietz and Woods 2002). The UPR levels were monitored by spectrofluorimetry (Xenius, SAFAS Monaco, Monaco) in yeast cells cultivated on agar plates at exponential phase growth, is to say, picked up when the areas of colonies were 0.25 ± 0.05 mm^2^. A synchrone GFP-detecting spectrum from 430 to 530 nm was obtained. The fluorescence was normalized with the number of cells given by the *A*_600 nm_. At least three biological repetitions were done for each experiment.

### RT-PCR and real-time RT-PCR

Experiments were performed on yeast cells cultivated on agar plate and treated or not treated with 20 mmol/L ZnSO_4_. The cells were harvested during their exponential growth phase. Total RNA extractions were performed with Trizol reagent (Gibco BRL, Life Technologies), purified by isopropanol precipitation then with RNeasy kit (Qiagen SAS, Courtaboeuf, FRANCE). Quality and quantity of RNA were controlled at each step by spectrometry (NanoDrop 1000; Thermo Scientific, Villebon sur Yvette, France). One micro gram of total DNA-Free RNA was reverse transcribed using M-MLV Reverse Transcriptase, RNase H Minus, Point Mutant (M3681; Promega, Charbonnieres, France) and 2 *μ*g of Oligo(dT)_15_ primer (C1101; Promega) in a final volume of 50 *μ*L, according to the manufacturer's instructions. To test *HAC1* mRNA splicing, a RT-PCR was performed according to Gardarin et al. (2010) with 1 *μ*L of cDNAs and 20 PCR-cycles. The primers used are given in Data S4.1. The PCR products were analyzed by gel electrophoresis (0.5% TAE, 1% agarose, ethidium bromide).

To evaluate the expression levels of *KAR2* and *HRD1* genes, Real-Time RT-PCRs were performed in 384-well plates with the LightCycler® 480 Real-Time PCR System (Roche Diagnostics GmbH, Meylan, France) using SYBR Green to monitor cDNA amplification. *ACTIN* and *TUBULIN* were used as internal controls for normalization. For each gene, specific primer pairs were designed (Data S4.2) and the PCR efficiency (E) of each primer pair was determined after the analysis of five serial 1:20 dilutions of cDNA. Relative expression ratios of target transcripts were then calculated according to Pfaffl method (Pfaffl 2001). For each treatment, three biological experiments were done. The Student's *t*-tests were performed to compare the expression levels of the different conditions.

## Results

### Production of a fully oxidized/folded recombinant AhPDF1.1b in *Pichia pastoris*

**Figure 1:**
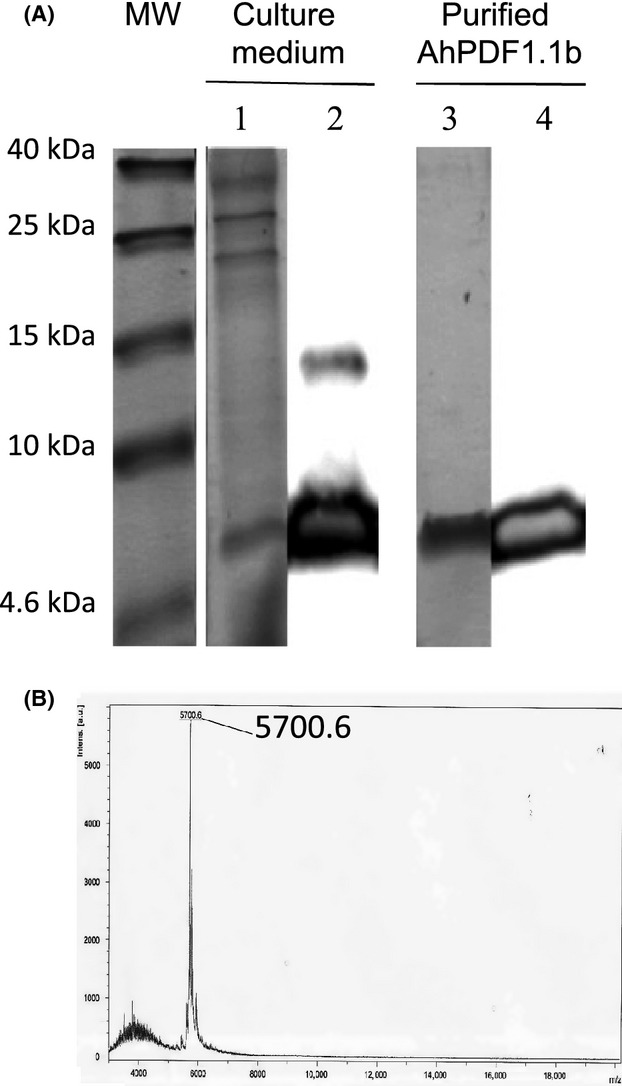
Production and purification of recombinant AhPDF1.1b in *Pichia pastoris*. (A) Analysis of the culture medium and the purified fraction of AhPDF1.1b produced in *P. pastoris*, on 18% (w/v) acrylamide ethylene-glycol SDS-PAGE. MW, molecular weight markers; Lane 1, culture medium, Coomassie staining. Lane 2, culture medium, western blot with anti-AhPDF1.1b polyclonal serum. Lane 3, final purified recombinant AhPDF1.1b, Coomassie staining. Lane 4, final purified recombinant AhPDF1.1b, western blot with anti-AhPDF1.1b polyclonal serum. (B) MALDI-TOF mass spectrum of the purified recombinant AhPDF1.1b produced in *P. pastoris*.

To test the activities of AhPDF1.1b when applied exogenously, it was necessary to produce and purify a native-like recombinant protein. In a previous study, we described the production of a native-like AhPDF1.1b in *E. coli* (Marquès et al. 2009). This protocol required an in vitro oxidation/refolding step, which could produce misfolded forms. In the present study, we tested a new production system using *P. pastoris*. The expression of several plant defensins in *P. pastoris* has already been reported (Kristensen et al. 1999; Almeida et al. 2001; Chen et al. 2004; Allen et al. 2008). This eukaryotic production system allowed us to directly obtain an in vivo fully oxidized/folded defensin. We chose a *Pichia* clone in which the KEX2 cleavage after the Glu-Lys-Arg residues has been efficient and produced native AhPDF1.1b without extra amino acids at its N-terminus. We used an optimized production in bioreactor (Brunel et al. 2004) that generated a high final biomass (*A*_600 nm_ = 300) and a high quantity of secreted proteins reaching 2 g.L^−1^. However, the subsequent purification step using cation exchange chromatography, as usually reported for plant defensin purification (Cabral et al. 2003; Chen et al. 2004; Allen et al. 2008), still needs to be improved in our hands to take full advantage of this very high production yield. Indeed, we observed that multimeric forms or aggregates of AhPDF1.1b appeared in the culture medium and likely prevented a high purification yield. Finally, around 1 mg of recombinant AhPDF1.1b was purified to homogeneity from a 1 L bioreactor as shown by SDS-PAGE electrophoresis and MALDI-TOF MS (Fig.1). The molecular mass of the purified recombinant AhPDF1.1b (5700.6 Da) was consistent with a fully oxidized form with four disulfide bonds formed (5701 Da expected).

### Recombinant AhPDF1.1b is a morphogenic antifungal protein

**Figure 2:**
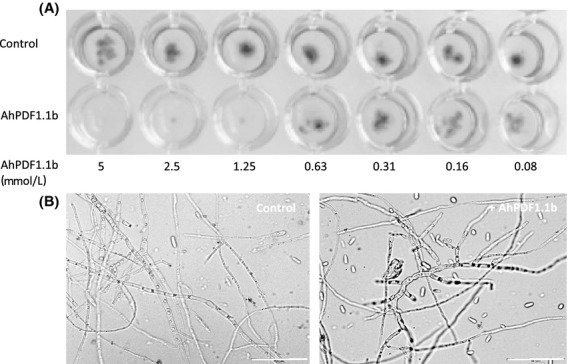
Growth inhibition assay on *Fusarium oxysporum* sp. *melonii*. (A) Twofold serial dilutions of purified recombinant AhPDF1.1b were tested on a spore suspension of *F. oxysporum* sp. *melonii* in half strength Potato Dextrose Broth and incubated 72 h at 30°C in darkness. (B) The morphology of the fungal hyphae has been observed after 36 h of growth of a spore suspension of *F. oxysporum* sp. *melonii* in half strength potato dextrose broth without any addition (control) or with the addition of 0.8 *μ*mol/L AhPDF1.1b. Bars correspond to 50 *μ*mol/L.

The recombinant AhPDF1.1b protein produced in *P. pastoris* showed an antifungal activity against *F. oxysporum* sp. *melonii* similar to that observed previously for AhPDF1.1b produced in *E. coli* (Marquès et al. 2009). The minimum inhibitory concentration measured was ∽1 *μ*mol/L (Fig.2A). We tested its activity against the *P. pastoris* yeast itself and no toxicity was found (data not shown). Previously, the *E. coli* recombinant defensin was found to be not toxic for *S. cerevisiae* or *Candida albicans* at concentrations as high as 20 *μ*mol/L (Marquès et al. 2009). In the present study, we also tested the capacity of the AhPDF1.1b defensin to cause a morphogenic growth inhibition that has not been assayed before for this protein. When observing the *Fusarium* hyphae grown in the presence of AhPDF1.1b, a clear hyperbranching was observed similar to what has already been described for other *Brassicaceae* defensins (Osborn et al. 1995) (Fig.2B).

### AhPDF1.1b inhibits root growth of *Arabidopsis thaliana* plantlets

**Figure 3:**
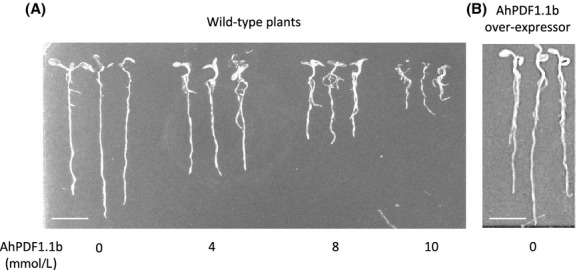
Inhibitory effect of AhPDF1.1b defensin on root growth. (A) Wild-type *Arabidopsis thaliana* plantlets grown for 8 days in a culture medium supplemented with concentrations of purified recombinant AhPDF1.1b ranging from 0 to 10 *μ*mol/L. (B) *A. thaliana* overexpressing AhPDF1.1b plantlets (Mirouze et al. 2006) grown for 8 days in a basal culture medium. Bar corresponds to 1 cm.

A toxic effect on plants themselves, namely inhibition of root growth, has previously been reported (Allen et al. 2008). A similar test was conducted with the recombinant AhPDF1.1b. When recombinant AhPDF1.1b was added in the culture medium of *A. thaliana* plantlets, a clear root growth inhibition was observed compared to the control without defensin (Fig.3A). This growth inhibition was dose dependent and quite similar to what has been already described for MsDef1, MtDef2, and RsAFP2 defensins with an active dose from 4 *μ*mol/L (Allen et al. 2008). The AhPDF1.1b defensin is thus another plant defensin showing a toxic effect on root growth when added to the culture medium. It is to be noticed that *A. thaliana* overexpressing AhPDF1.1b that we previously produced (Mirouze et al. 2006) did not show any growth inhibition compared to wild-type plants (Fig.3B).

### When expressed in cells, AhPDF1.1b confers zinc tolerance independently of ZRC1

**Figure 4:**
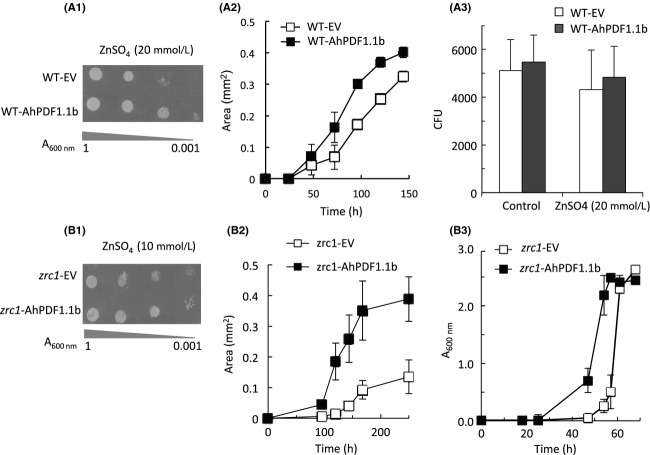
Zn tolerance phenotypes of wild-type yeast expressing *AhPDF1.1b* (A) or *zrc1* mutant expressing *AhPDF1.1b* (B). (A.1) Drop-test assays of wild-type yeast expressing the pYX212 empty vector (WT-EV) or wild-type yeast expressing AhPDF1.1b (WT-AhPDF1.1b) on 20 mmol/L ZnSO_4_ agar plates. An overnight yeast culture was diluted to the *A*_600nm_ levels indicated and 10 *μ*L were spotted on YNB medium supplemented or not with 20 mmol/L ZnSO_4_. (A.2) Growth curves of wild-type yeast expressing the pYX212 empty vector (WT-EV) or wild-type yeast expressing AhPDF1.1b (WT-AhPDF1.1b) plated on agar medium supplemented with 20 mmol/L ZnSO_4_. Isolated colonies plated on 20 mmol/L ZnSO_4_ were photographed with a SZX16 Olympus stereozoom microscope and their areas were measured using ImageJ software. Error bars correspond to standard errors, *n* ≥ 50. (A.3) Number of isolated colonies (CFU) of wild-type yeast expressing the pYX212 empty vector (WT-EV) or wild-type yeast expressing AhPDF1.1b (WT-AhPDF1.1b) plated on agar medium supplemented with 20 mmol/L ZnSO_4_. The number of colonies was counted after plating 100 *μ*L of a *A*_600nm_ = 0.01 culture of yeast cells. The Student's *t*-tests give a *P* = 0.47 on the control medium and a *P* = 0.27 on the zinc-supplemented medium. Drop-test assays of *zrc1* yeast expressing the empty vector (*zrc1*-EV) or *zrc1* yeast expressing AhPDF1.1b (*zrc1*-AhPDF1.1b) on 10 mmol/L ZnSO_4_ agar plates. An overnight yeast culture was diluted to the *A*_600nm_ levels indicated and 10 *μ*L were spotted on YNB medium supplemented or not with 10 mmol/L ZnSO_4_. B.2 Growth curves of *zrc1* yeast expressing the empty vector (*zrc1*-EV) or *zrc1* yeast expressing AhPDF1.1b (*zrc1*-AhPDF1.1b) obtained by measuring the areas of isolated colonies plated on 10 mmol/L ZnSO_4_. Error bars correspond to standard errors, *n* ≥ 50. B.3 “Zinc shock” assay in liquid culture of *zrc1* yeast expressing the empty vector (*zrc1*-EV) or *zrc1* yeast expressing AhPDF1.1b (*zrc1*-AhPDF1.1b). The growth curves are followed by *A*_600nm_ measurements during a zinc shock assay as described in [27]. Error bars correspond to standard errors, *n* = 3.

Expression of the AhPDF1.1b defensin (including its signal peptide) in wild-type *S. cerevisiae* yeast was previously shown to confer zinc tolerance in drop-test assays (Fig.4A.1 and Mirouze et al. 2006). To check whether AhPDF1.1b improved the growth rate or the survival of yeast cells during zinc overload, which is difficult to evaluate using drop-test assays, yeast culture was plated at a low density on a zinc-supplemented agar plate to follow both the area of colonies and their number. On plates supplemented with zinc, wild-type yeast cells expressing AhPDF1.1b displayed an increase in the colony areas (Fig.4A.2) but the same number of colonies (Fig.4A.3) compared to wild-type yeast expressing the empty vector. When 20 mmol/L of ZnSO_4_ was added to the culture medium, the cell survival of yeast was not altered and the expression of AhPDF1.1b improved the growth rate. Following the increase in the area of colonies over time, we could plot a growth curve of yeast cultivated on agar plates and assess growth over time under different conditions what was not possible with drop tests. The zinc tolerance phenotype already reported in drop-test assays was clearly observed during the exponential growth phase of wilt-type yeast expressing AhPDF1.1b (Fig.4A.2).

Unexpectedly, AhPDF1.1b was unable to improve zinc tolerance of yeast cells when they were grown in liquid culture. We decided to test the “zinc shock” assays worked out by MacDiarmid in liquid cultures (MacDiarmid et al. 2003). In this assay, the *zrc1* mutant, impaired in the ZRC1 vacuolar zinc transporter, is used. This mutant is no longer able to rescue the cytoplasm by loading the vacuole what allows to perform a “zinc shock” when zinc-depleted *zrc1* cells are resupplied with zinc. The wild-type yeast is not sensitive to “zinc shock” (MacDiarmid et al. 2003) and this assay can only be performed with the *zrc1* mutant strain, which is more sensitive to zinc than wild-type yeast. We first tested the *zrc1* mutant on drop-test assays and showed that AhPDF1.1b expression was able to confer a zinc tolerance phenotype on 10 mmol/L ZnSO_4_ plates in this genetic background (Fig4B.1). With plating test, it clearly appeared that the expression of AhPDF1.1b in this mutant deeply enhanced its tolerance to zinc (Fig.4B.2). With the “zinc shock” assay, it has even been possible to observe a zinc tolerance in liquid culture (Fig.4B.3). It was the first assay performed in liquid culture in which we observed a zinc tolerance phenotype conferred by AhPDF1.1b. These results showed that in the *zrc1* mutant background the defensin conferred a high zinc tolerance phenotype. The ZRC1 transporter, which is the main tonoplastic transporter responsible for zinc sequestration in the vacuole, is thus not implicated in the mode of action of AhPDF1.1b to confer zinc tolerance.

### AhPDF1.1b confers Zn tolerance from the endoplasmic reticulum

**Figure 5:**
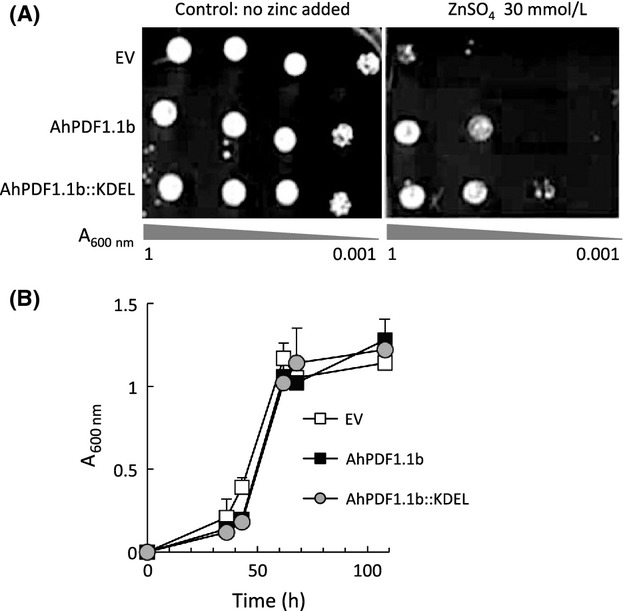
Zinc tolerance assay of yeast expressing *AhPDF1.1b::KDEL*. (A) Drop tests were performed with control yeast cells expressing the pYX212 empty vector (EV), or the native AhPDF1.1b (AhPDF1.1b) or a recombinant AhPDF1.1b defensin fused with the KDEL retention signal at its C-terminal end (AhPDF1.1b::KDEL). An overnight yeast culture was diluted to the *A*_600nm_ levels indicated and 10 *μ*L were spotted on YNB medium supplemented or not with 30 mmol/L ZnSO_4_. (B) Growth curves in liquid cultures contaminated by 20 mmol/L ZnSO_4_, of control yeast cells expressing the pYX212 empty vector (EV), or the native AhPDF1.1b (AhPDF1.1b) or a recombinant AhPDF1.1b defensin fused with the KDEL retention signal at its C-terminal end (AhPDF1.1b::KDEL). The growth was followed by *A*_600nm_ measurement.

We previously showed that AhPDF1.1b is not secreted when expressed in yeast but rather accumulated in the endoplasmic reticulum (ER) and prevacuolar compartments (Oomen et al. 2011). To block the defensin in the ER compartment, a construct enabling the translational fusion of the ER-targeting motif KDEL downstream of the AhPDF1.1b was engineered. KDEL motif at the C-terminal end of a protein is the classical ER-retention signal (Pichon et al. 1997) usually used to engineer ER resident proteins (Young et al. 2013). Yeast cells expressing the AhPDF1.1b::KDEL fusion displayed an even greater zinc tolerance activity than yeast cells expressing the native AhPDF1.1b compared to WT cells (Fig.5A). This result suggests that AhPDF1.1b confers zinc tolerance when it is blocked in the ER. In liquid culture, the AhPDF1.1b::KDEL fusion did not confer any zinc tolerance and show the same growth curve as yeast expressing the empty vector or yeast expressing AhPDF1.1b (Fig.5B).

### When expressed in cells, AhPDF1.1b confers tolerance to diamide but not to H_2_O_2_ or menadione

**Figure 6:**
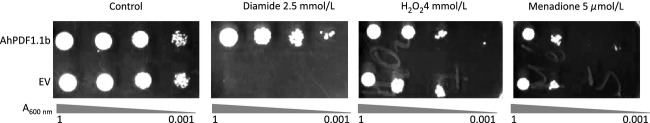
Tolerance assays in response to various oxidative treatments. Drop tests were performed with wild-type yeast cells expressing the native AhPDF1.1b (AhPDF1.1b) and control yeast expressing the pYX212 empty vector (EV). Yeast cells were cultivated overnight and then diluted to the *A*_600nm_ levels indicated. About 10 *μ*L were spotted onto YNB medium (control) or onto YNB supplemented with 2.5 mmol/L diamide or 4 mmol/L H_2_O_2_ or 5 *μ*mol/L menadione.

Protein oxidation/folding is one of the major functions of the ER. As a cysteine-rich protein with four disulfide bridges, AhPDF1.1b is oxidized/folded in the ER when expressed in yeast. We assayed three oxidizing agents: diamide (diazine-dicarboxylic acid-bis(dimethylamide), H_2_O_2_*,* and menadione to evaluate the influence of the AhPDF1.1b expression on different redox stresses. H_2_O_2_ and menadione modify both reduced/oxidized glutathione (GSH/GSSG) ratio and intracellular reactive oxygen accumulation, while diamide decreases specifically the GSH/GSSG ratio without altering peroxide and superoxide anion levels (Pocsi et al. 2005). GSH/GSSG ratio is crucial for ER redox status and thereby diamide specifically perturbs the redox status of the ER. It has been shown that diamide toxicity is mediated in part by disruption of disulfide isomerisation in the ER and associated with vacuolar protein-sorting functions (Thorpe et al. 2004). In drop-test assays, AhPDF1.1b appeared to protect yeast cells from the toxicity of diamide but not from that of the two other oxidizing agents (Fig.6). This result is fully consistent with a role of AhPDF1.1b in the ER and prevacuolar compartments.

### AhPDF1.1b expression in yeast cells triggers the quality control system of the ER

**Figure 7:**
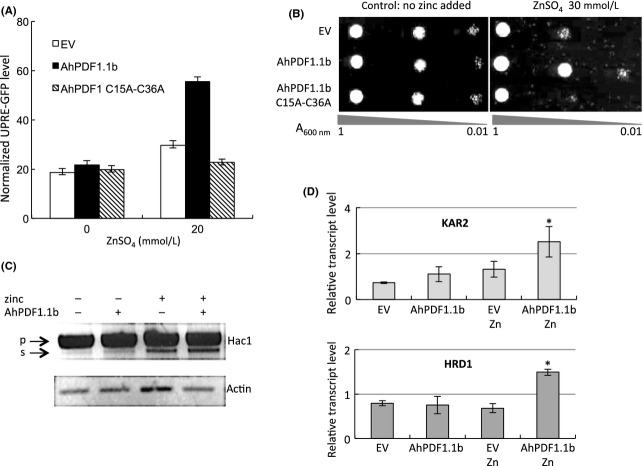
Synergistic effect of zinc overload and AhPDF1.1b expression on UPRE activation and ERAD. (A) Expression of the UPRE-GFP reporter system in yeast cells expressing the pFL38H empty vector (EV) or the native AhPDF1.1b (AhPDF1.1b) or the mutated C15A-C36A AhPDF1.1b (AhPDF1.1b C15A-C36A). The GFP levels were acquired through a synchrone spectrum from 430 to 530 nm. Yeast cells were cultivated on agar plates with increasing zinc concentrations and picked up on their exponential growth phase (see Fig.4A.2). The fluorescence was normalized by the number of cells given by the *A*_600nm_. At least three biological repetitions were done for each experiment. Error bars correspond to standard errors, *n* = 3. (B) Drop-test performed with yeast cells expressing the pFL38H empty vector (EV) or the native AhPDF1.1b (AhPDF1.1b) or the mutated C15A-C36A AhPDF1.1b (AhPDF1.1b C15A-C36A). An overnight yeast culture was diluted to the *A*_600nm_ levels indicated and 10 *μ*L were spotted on YNB medium supplemented or not with 30 mmol/L ZnSO_4_. (C) *HAC1* mRNA splicing in yeast cells expressing or not the native AhPDF1.1b and grown on agar plates supplemented or not with 20 mmol/L zinc. After total RNAs extraction, *HAC1* mRNA splicing was monitored by RT-PCR. PCR products were separated by electrophoresis on a 1% agarose gel. The positions of the bands corresponding to the precursor (p) or the spliced (s) *HAC1* mRNA are indicated. (D) The expressions of *KAR2* and *HRD1* were monitored by Real-time RT-PCR. mRNA were isolated from yeast expressing the pYX212 empty vector (EV) or expressing the native AhPDF1.1b (AhPDF1.1b) and grown on control conditions or in the presence of 20 mmol/L ZnSO_4_ (Zn). Three biological replicates were analyzed and transcript quantifications were expressed relatively to the expression of two housekeeping genes: *ACTIN* and *TUBULIN*. Error bars correspond to standard errors, *n* = 3. The asterisk (*) indicated Student's *t*-test *P *≤ 0.05 when compared AhPDF1.1b-Zn with the other conditions.

To test for an effect of AhPDF1.1b expression on ER-stress, a reporter system, based on a transcriptional fusion between a synthetic UPRE and a GFP gene, was used (Jonikas et al. 2009). This UPRE promoter was composed of four repeats of the *KAR2* UPRE. The zinc treatment alone led to increased GFP expression driven by the UPRE reporter promoter. This response was twofold higher when AhPDF1.1b was expressed in yeast during the zinc overload. The expression of AhPDF1.1b alone did not trigger any UPRE-GFP reporter response (Fig.7A). Thus, a clear synergistic effect of both zinc and defensin expression drove the UPRE-reporter response. A mutated AhPDF1.1b in which the Cys_15_ and the Cys_36_ had been replaced by alanine preventing the formation of one of the four disulfide bonds was neither able to confer zinc tolerance (Fig.7B) nor to trigger the UPRE-GFP reporter response (Fig.7A).

The UPRE is a binding site for the Hac1 transcription factor. In response to ER-stress, Hac1 activity is controlled at the level of mRNA splicing. The *HAC1* splicing is induced by misfolded proteins appearing in the ER and the spliced product is the transcription factor able to drive the activity of the reporter system. Therefore, *HAC1* splicing was analyzed in response to zinc and AhPDF1.1b expression. Zinc overload alone triggered a clear splicing of the *HAC1* mRNA while the expression of AhPDF1.1b alone had little effect (Fig.7C). These results are consistent with those obtained with the reporter system. However, the synergistic effect of defensin expression and zinc overload on *HAC1* splicing was not clearly observed. Therefore, to confirm the results obtained with the reporter system, we carried out real-time RT-PCR to analyze *KAR2* and *HRD1* expression in response to both zinc overload and defensin expression. As previously mentioned, *KAR2* promoter contains the UPRE used in the reporter system, and *HRD1* gene is an UPR-responsive gene, involved in ERAD (ER-associated degradation). *KAR2* and *HRD1* were both found to be upregulated specifically in zinc plus defensin conditions compared to control condition, or to zinc overload alone or to expression of AhPDF1.1b alone (Fig.7D). These data support the synergistic effect of zinc and AhPDF1.1b in triggering the expression of genes known to be involved in the ER quality control system.

## Discussion

In the present study, a comprehensive analysis of the different activities of the AhPDF1.1b defensin was conducted through an original approach consisting both (1) in producing recombinant AhPDF1.1b to explore its various roles when added externally and (2) in expressing AhPDF1.1b in yeast to confer zinc tolerance. AhPDF1.1b had been previously shown not to be secreted when it is expressed in yeast (Oomen et al. 2011) and thereby the second point deals with a bioactivity exerted from the inside of the cells, which is quite new for a defensin study. With this experimental design, we could demonstrate that AhPDF1.1b has quite divergent bioactivities depending on its localization: applied exogenously or expressed and retained inside the cell.

### Recombinant AhPDF1.1b is a toxin when applied exogenously

All the 12 3D structures of plant defensins available in the RCSB Protein Data Bank (http://www.rcsb.org/pdb/home/home.do) harbor a globular cysteine-stabilized *α*-helix *β*-sheet (CS*αβ*) structure with four disulfide bonds (Cornet et al. 1995). All these defensins which are extracted from plant tissues (seeds or flowers) are fully oxidized and their 3D structures have been solved in this conformation. Although they show a great variability in protein sequence, their 3D structure is highly conserved and gives them a highly resistant scaffold (Gachomo et al. 2012). To obtain a native-like folded recombinant defensin, we chose the *Pichia* production system. Indeed, *E. coli* and *P. pastoris* are quite different expression systems that produce denatured or fully oxidized folded defensin, respectively. When produced in *E. coli*, the recombinant AhPDF1.1b is not soluble and must be extracted with a chaotropic agent (guanidinium chloride) from the inclusion bodies (Marquès et al. 2009). A renaturation step is then required to obtain the folded protein. This step is nonspecific and leads to various conformations of the protein. In contrast, in the eukaryotic organism *P. pastoris*, in vivo processing and folding of the recombinant protein occur and produce a secreted oxidized/folded protein with the expected four disulfide bonds. The fully oxidized recombinant AhPDF1.1b obtained through *Pichia* production is a canonical antifungal plant defensin quite similar to RsAFP2 (identity 90.2%; similarity 98%). AhPDF1.1b clusters with all the *Brassicaceae* defensins in the group 9 of the phylogenetic classification recently published (van der Weerden and Anderson 2013). We showed in the present study that it is a morphogenic antifungal defensin such as RsAFP2 and almost all the defensins from this group. This result confirms the conclusion of van der Weerden's study on the interest of such a clustering for the prediction of some defensin properties according to their phylogenetic group.

We then asked if AhPDF1.1b could inhibit the root growth as the MsDef1 and Mtdef2 defensins do (Allen et al. 2008). These defensins are classified into the group 10 of van der Werdeen's clustering, thus in another phylogenetic group but rather close to group 9. We found exactly the same property for AhPDF1.1b as for the MsDef1 and MtDef2 defensins. It thus appears that plant defensins from different phylogenetic groups are active against plant cells themselves exerting a toxic action on their development. Until now, only a toxic action on fungal cells was generally considered. When a recombinant defensin is produced and applied externally to fungal or plant cells it thus behaves as a toxin. This is a quite important result to keep in mind if plant defensins are to be used as antifungal agents.

### AhPDF1.1b expressed in yeast cell confers zinc tolerance by triggering an adaptive response in the ER

We previously showed that the expression of AhPDF1.1b in yeast and plant cells confers a tolerance to zinc overload (Mirouze et al. 2006; Shahzad et al. 2013). This phenotype was quite unexpected since it was the first time that a defensin was described not only as a toxin but also as a positive factor on growth. This zinc tolerance phenotype was described in yeast on solid plate and was not detected in liquid culture. In the present study, we tried to characterize more accurately this phenotype in yeast and we showed that the defensin expression actually increases the growth rate of yeast. The further characterization of the zinc tolerance thanks to the use of the *zrc1* mutant in the “zinc shock” protocol (MacDiarmid et al. 2003) revealed that AhPDF1.1b was able to confer tolerance to zinc to the *zrc1* mutant in liquid culture. ZRC1 is the main tonoplastic zinc transporter involved in the loading of the vacuole (MacDiarmid et al. 2002) and as such, it is essential to overcoming the zinc shock (the zinc shock is anyway not possible in the wild-type yeast with a functional ZRC1). This result prompted us to hypothesize that AhPDF1.1b was able to counteract a cytoplasmic zinc overload without the involvement of the buffering action of the vacuole. The protection of the cell mediated by AhPDF1.1b could, therefore, occur through another mechanism.

To understand the mode of action of AhPDF1.1b in zinc tolerance, one key point is the localization of the defensin when it is expressed in yeast. We previously showed that AhPDF1.1b expressed in yeast or in leaves is not secreted but accumulated in internal compartments (Oomen et al. 2011). AhPDF1.1b is targeted to the secretory pathway via the ER, but it does not follow the entire route to the plasma membrane and is retained inside the cell. We found here that if AhPDF1.1b is artificially retained in the ER by a KDEL sequence, the zinc tolerance phenotype is conserved. On the other hand, we tested various oxidizing treatments and we found that AhPDF1.1b expression only confers a tolerance to the diamide treatment. Diamide is a disulfide-stress agent known to interfere specifically with protein folding in the ER (Leichert et al. 2003). AhPDF1.1b can thus exert its beneficial action as soon as it is in the ER. This finding is quite important since there is a growing consensus to forward the idea that unexpected activities must exist for defensins inside the organisms that produce them.

Zinc is an essential element for ER function (Ellis et al. 2004). Zinc deficiency was shown to trigger an ER-stress and the UPR in yeast (North et al. 2012). However, nothing is known for zinc overload. All our data prompted us to explore the role of the cysteine-rich AhPDF1.1b on ER-stress during zinc overload. By using an UPRE-GFP reporter system, we found a synergistic effect of AhPDF1.1b expression and zinc overload in triggering the UPRE-driven expression of GFP. This response was abolished in the mutated C15A-C36A AhPDF1.1b showing that these cysteines were important for this activity. The spliced form of *HAC1*, which is the transcription factor driving the UPRE*,* was detected during zinc overload but the synergistic effect of zinc and defensin expression was not clearly observed and the splicing was always partial. Usually, *HAC1* splicing is a transient phenomenon studied in liquid culture. In our conditions on agar plates, it is possible that we did not pick the optimal time to observe differences in splicing. Consistent with the results obtained with the GFP-reporter system, a synergistic effect of the AhPDF1.1b expression and zinc overload was observed on the expression levels of *KAR2* and *HRD1* which are two Hac1-target genes. Our data thus argue in favor of the occurrence of an adaptive response of yeast expressing AhPDF1.1b that leads to improve the cell fitness during the zinc overload. Overall these results suggest that AhPDF1.1b could contribute to the protein quality control to mitigate ER-stress. We hypothesize that inside the ER, the AhPDF1.1b defensin, through its numerous cysteines, could interfere with zinc homeostasis and/or protein folding. It could be that the protein is not fully oxidized in this compartment and directly interact with zinc itself or with some zinc-requiring protein. Recently an interaction between zinc and a mammal defensin has been reported (Zhang et al. 2013). We are currently exploring this hypothesis by studying the redox status of the different disulfide bridges of AhPDF1.1b to know if they could be reduced in the ER and interact with zinc homeostasis of this compartment.

## Conclusion

When defensins are released from the cell they are found fully oxidized and are toxic to both fungal and plant cells altering both growth and development. Although these activities of plant defensins have been well known for a long time, the mode of action is still unclear and probably not unique (Maroti et al. 2011; Munoz et al. 2014). Up to now, very little is known about the role of defensins inside the organisms that produce them. We showed in this study that plant defensins when they are retained inside the cell are no more toxic but instead can improve stress tolerance. AhPDF1.1b seems to play a role in ER-stress and improve cellular adaptive response to zinc overload. Overall these results provide a better understanding of the role of plant defensins, these multifunctional proteins which are under an active divergent selection, highly duplicated in genomes, and on which we have still a lot to discover.

## Supporting Information

**Data S1** Codon optimization of AhPDF1.1b sequence by SOEing PCR-based method (Vallejo et al.1994). **Data S2** SDS-PAGE and western-blot and Maldi-TOF analyses. **Data S3** List of the primers used for the replacement of Cys15 and Cys36 codons in alanine codon. **Table S1** List of the primers used in RT-PCR and real-time RT-PCR.
