# Supplementary material for: The antifungal plant defensin AhPDF1.1b is a beneficial factor involved in adaptive response to zinc overload when it is expressed in yeast cells
Source: Microbiologyopen. 2015 Mar 8;4(3):409–22. doi: 10.1002/mbo3.248 (PMC4475384; doi:10.1002/mbo3.248)
Supplement: Supplementary file 1 — Data S1 Codon optimization of AhPDF1.1b sequence by SOEing PCR-based method (Vallejo et al.1994). Data S2 SDS-PAGE and western-blot and Maldi-TOF analyses. Data S3 List of the primers used for the replacement of Cys15 and Cys36 codons in alanine codon. Table S1 List of the primers used in RT-PCR and real-time RT-PCR. [file mbo30004-0409-sd1.pdf]

SUPPLEMENTARY DATA

The antifungal plant defensin AhPDF1.1b is a beneficial factor involved in adaptive response to zinc overload when it is expressed in yeast cells

Mith O[1], Benhamdi A[1], Castillo T[1], Bergé M[1], MacDiarmid CW[2], Steffen J[2], Eide DJ[2], Perrier V[3], Subileau M[3], Gosti F[1], Berthomieu P[1], Marquès L[1]

Corresponding author: Laurence Marquès
Montpellier SupAgro / Université Montpellier 2 / INRA / CNRS UMR B&PMP Biochimie et Physiologie Moléculaire des Plantes, Campus Montpellier SupAgro, 2 Place Viala, F-34060 Montpellier Cedex 2, France.
phone : +33 499 613 157, fax : +33 467 525 737, mail : laurence.marques@univ-montp2.fr

Supplementary data 1

Codon optimization of AhPDF1.1b sequence by SOEing PCR-based method (Vallejo et al., 1994)

The initial sequence of the mature AhPDF1.1 was:

5'- CAGAGGTTGTGCGAGAAGCCAAGTGGGACATGGTCAGGAGTTTGCGGAAACAATGGCGCG

TGCAGAAATCAGTGCATTAGACTTGAGAAAGCACGACATGGATCTTGCAACTACGTCTTCCCAGCT

CATAAGTGTATCTGTTACTTCCCATGTTAA

and was transformed to :

5'-CAG*AGA*TTG*TGTGAA*AAGCCA*TCTGGTACT*TGG*TCTGGT*GTT*TGTGGT*AACAAT*GGTGCT*TGCAGA

AATCAGTGCATTAGACTTGAGAAA*GCTAGA*CAT*GGT*TCT*TGT*AACTAC*GTTTTT*CCAGCTCATAAGTG

T*ATT*TGTTAC*TTT*CCATGTTAA

using the following primers :

5'-GGG CTC GAG AAA AGA CAG AGA TTG TGT GAA AAG

5'-AGA ACC ATG TCT AGC TTT CTC AAG TCT AAT G

5'-GCT AGA CAT GGT TCT TGT AAC TAC GTT TTT CCA GCT CAT AAG TGT

5'-TGT TCT AGA TTA ACA TGG AAA GTA ACA AAT ACA CTT ATG AGC TGG

Supplementary data 2

SDS-PAGE and Western-blot

Samples were mixed with loading buffer 5X (200 mM Tris-HCl, pH 6.8; 10% sodium dodecyl sulphate (SDS); 5% β-mercaptoethanol; 0.1% bromophenol blue) and boiled for 5 min before loading on a 18% SDS-PAGE containing ethylene-glycol (ethylene-glycol 30% ; acrylamide 19 :1 18%, Tris 0.75 M pH 8.5, ammonium persulfae 0.05%, TEMED 0.1%)

For western-blotting, samples were subsequently transferred onto polyvinyldifluoride Immobilon-P^{SQ} membrane (Merck-Millipore, Darmstadt, Germany) by liquid transfer technique. Anti-AhPDF1.1b polyclonal antibodies raised in rabbit against the AhPDF1.1b protein produced in *E. coli* according to Marquès et al. (2009) were used as primary antibodies. Membranes were blocked in Tris-buffered saline (TBS: 20 mM Tris-HCl, pH 7.5, 150 mM NaCl) supplemented with 5% dried milk and then incubated in TBS with 1% dried milk and anti-AhPDF1.1 (1 : 800) for 18 h at 22° C. Membranes were washed with TBST (TBS with 0.2% Tween 20) and incubated with anti-rabbit antibodies coupled with alkaline phosphatase (1 : 30000; Sigma) in TBST with 1% dried milk for 1 h at 22° C. After washing with TBS and 20 mM Tris- HCl with 1 mM $MgCl_2$, proteins were visualized on a LAS 3000 Imager (Fujifilm, Valhalla, NY, USA) after 10 min incubation with Lumi-Phos™ WB (Thermo Fisher Scientific, Waltham, USA).

Maldi-TOF analysis

MALDI-TOF mass spectra were acquired on Ultraflex (Bruker). The irradiation source was a pulsed nitrogen laser with a wavelength of 337 nm. The matrix solution (sinapinic acid at 10 mg ml)1) and fractions were mixed in a ratio of 10 : 1. One microliter of the mixture was deposited and air-dried. External mass calibration was performed with a standard protein mixture. Mass spectra were analysed with flexanalysis software (Bruker).

Supplementary data 3

Table 3.1 List of the primers used for the replacement of Cys15 and Cys36 codons in alanine codon

Primer name	Primer sequence [a]
ATG_forward	5‘-AAAGAATTCATGGCTAAGTTTGCTTCCATC
C15A_reverse	5’-CCATTGTTTCC**AGC**AACTCCTGACCATGTCC
C15A_forward	5’-CAGGAGTT**GCT**GGAAACAATGGCGCGTG
C36A_reverse	5’-AGACGTAGTT**AGC**AGATCCATGTCGTGCTTTC
C36A_forward	5‘-TGGATCT**GCT**AACTACGTCTTCCCAG
STOP_reverse	5’-CCCCTCGAGTTAACATGGGAAGTAACAG
[a] Restriction sites used for the cloning are underlined. Alanine codons are in bold.	

In order to change the Cys15 and Cys36 codons into alanine, two successive SOEING PCR were performed. First the Cys15 codon was changed using the ATG-forward primer together with the C15A-reverse primer on one side, and the C15A-forward primer together with the STOP-reverse primer on the other side. Then the Cys36 codon was changed in the C15A-AhPDF1.1b mutant using the ATG-forward primer together with the C36A-reverse primer on one side, and the C36A-forward primer together with the STOP-reverse primer on the other side.

Supplementary data 4:

Table 4.1 List of the primers used in RT-PCR

HAC1	5′-AGGAAAAGGAACAGCGAAGG
	5′-GAATTCAAACCTGACTGCGC
Actin 1	5′-GCTTTGTTCCATCCTTCTG
	5′-GAAACACTTGTGGTGAACG

Table 4.2 List of the primers used in real-time RT-PCR

KAR2	5’-AAGTCCAAGCCACTTCTGGT
	5’-ACATTTGGCTGGACAAGGCA
HRD1	5’-ACTGGGATAGCGACAGATCA
	5’-ATCGGCTTGACCCAACAGAA
Actin 1	5’-ATGGAGCCAAAGCGGTGATT
	5’-AGAGCCCCAGAAGCTTTGTT
Tubulin 1	5’-TTCTCTTCCAAGCCTCAGCA
	5’-AGCTGTCGAGCAGGTGAAAA
